# Recommendations on how to proceed in case of suspected allergy to penicillin/β-lactam antibiotics: Position paper of the German Society for Allergology and Clinical Immunology (DGAKI) in cooperation with the German Society for Pediatric Allergology (GPA), Austrian Society for Allergology and Immunology (ÖGAI) and the Swiss Society for Allergology and Immunology (SGAI)

**DOI:** 10.5414/ALX02531E

**Published:** 2025-03-06

**Authors:** Knut Brockow, Wolfgang Pfützner, Bettina Wedi, Gerda Wurpts, Axel Trautmann, Burkhard Kreft, Timo Buhl, Mathias Sulk, Andreas Recke, Kathrin Scherer, Stefan Wöhrl, Irena Neustädter, Regina Treudler, Christiane Querbach, Margitta Worm

**Affiliations:** 1 Department of Dermatology and Allergology Biederstein, Faculty of Medicine and Health, Technical University of Munich, Munich,; 2 Department of Dermatology and Allergology, University Hospital Giessen and Marburg, Marburg,; 3 Hanover Medical School, Department of Dermatology, Allergology and Venereology, Hanover,; 4 Department of Dermatology and Allergology, Germany, Aachen Comprehensive Allergy Center (ACAC), University Hospital of RWTH Aachen University, Aachen,; 5 Department of Dermatology and Allergology, Allergy Center Mainfranken, University Hospital Würzburg, Würzburg,; 6 Department of Dermatology and Venereology, University Hospital Halle, Halle (Saale),; 7 Department of Dermatology, Venereology and Allergology, University Medical Center Göttingen, Göttingen,; 8 Department of Dermatology, Münster University Hospital, Münster,; 9 Department of Dermatology, Allergology and Venereology, University Hospital Schleswig-Holstein, Holstein Campus Lübeck, Lübeck, Germany,; 10 Department of Dermatology, Cantonal Hospital Aarau, Aarau, and Department of Dermatology and Allergology, University Hospital Basel, Switzerland,; 11 Floridsdorf Allergy Center (FAZ), Vienna, Austria,; 12 Pediatric Clinic, Diakoneo Klinik Hallerwiese-Cnopfsche Kinderklinik, Nuremberg,; 13 Institute for Allergy Research, Charité Universitätsmedizin Berlin, Campus Benjamin Franklin, Berlin,; 14 Hospital Pharmacy, Klinikum rechts der Isar, Technical University of Munich, Munich, and; 15 Allergology and Immunology, Department of Dermatology, Venereology and Allergology, Charité-Universitätsmedizin Berlin, Berlin, Germany

**Keywords:** β-lactam antibiotics, penicillin allergy, risk stratification, allergy diagnostics, delabeling

## Abstract

β-lactam antibiotics (BLAs) are still the antibiotics of first choice for the treatment of many bacterial infections. Treatment with a BLA is often hindered by a suspected allergy, up to 10% of the population report an allergy to penicillin. After allergological evaluation of the suspected allergic reaction to a BLA, most patients show a low probability of a BLA allergy; only in a minority of cases an allergic reaction to the repeated administration of a BLA appear likely in view of the previous history. In > 90% of cases, the suspected BLA allergy can be ruled out by allergy diagnostics. We recommend a risk-stratified approach in the context of an urgent need for BLA, which should enable most patients to receive a BLA therapy. After acute therapy, allergy diagnostics have to be done to clearly prove or reliably rule out a BLA allergy.

## Introduction and methodology 

Due to an increasing number of inquiries, the working group Drug Allergy of the German Association of Allergology and Clinical Immunology (DGAKI) was commissioned to give recommendations on the procedure in cases of reported allergy to β-lactam antibiotics (BLAs). The reasons why adequate clarification of BLA allergy is important have been compiled and summarized in this position paper. A preliminary version was prepared and edited on the basis of standard operating procedures from various hospitals (MW, WP, BW, MS, AT, CQ, KB) and current publications. After internal adjustments by a core working group, it was presented first to further members of the DGAKI and then to experts from the other specialist societies with experience in the treatment of hypersensitivity reactions to BLAs. The draft text was revised according to the comments of all authors. Controversial points were clarified, and a final wording was consented. 

The recommendations formulated here are a consensus on how to proceed in cases with urgent indication for the administration of BLAs in the possible presence of a BLA allergy. In the meantime, numerous other algorithms and risk categories have been published, but these are defined heterogeneously in the literature and have been studied almost exclusively in monocentric studies on smaller collectives [6, 17, 18, 19, 20, 21, 22, 23]. The simplest algorithm (PenFast) classifies the risk of future BLA administration in patients with a history of BLA allergy on the basis of just four questions. Direct administration of BLAs with a low risk assessment was evaluated as safe in one study (but with an extremely low reaction rate) [20]. However, the specific rate of safety must first be confirmed in larger studies before this algorithm can be recommended for general practice due to its simplicity. It is undisputed that there is an increased risk of a new reaction in patients who have already suffered an objective and medically documented reaction to a BLA. Fatal reactions have also been described in individual cases [26]. 

## Importance of BLAs 

Penicillins, cephalosporins, carbapenems, monobactams, and β-lactamase inhibitors are BLAs. Due to their favorable benefit/risk profile, they are the first choice for the treatment of numerous bacterial infections such as respiratory, urinary tract, skin, soft tissue, and implant-associated infections [1]. “Penicillin” is often used as a synonym for all BLAs, sometimes even for all antibiotics [2]. 

## Epidemiology of suspected BLA allergy 

BLAs are used very frequently. They are the most commonly reported elicitors of drug allergy [3]. Depending on the country, the prevalence of BLA allergy in the general population is estimated at ~ 5 – 10%. In hospitalized cases, it is slightly higher at 10 – 25% [3, 4]. Even a vague suspicion, for example anecdotal information about a “skin rash” after BLA administration in childhood, often leads to a label of “penicillin allergy” [5, 6]. However, only a small proportion of those with “penicillin allergy” label, often after severe anaphylaxis, have a high probability of a true BLA allergy based on their medical history [2]. In over 90% of those who reported a penicillin allergy, allergy diagnostics, including provocation tests, were unable to prove it [3, 6]. This means that ~ 1% of the population and 2% of inpatients suffer from BLA allergy [3, 6]. 

## Consequences of avoiding BLAs 

Suspected BLA allergies lead to lifelong avoidance of all BLAs for fear of severe allergic reactions. Second-choice antibiotics are used as an alternative [4, 6]. This approach can be a considerable disadvantage for those affected (Table 1). Therefore, a suspected BLA allergy should not be accepted uncritically and should always be clarified by an allergologist [2]. 

## Simplified classification of BLA hypersensitivity reactions 

Drug hypersensitivity reactions are classified according to the time course, the clinical picture, and the underlying pathomechanism [2, 7, 8, 9]: 

### 1. Chronology 

After exposure to the BLA, immediate reactions occur within minutes to usually 1 hour after the first administration, rarely up to 6 hours. Delayed reactions occur after several hours (> 6 hours) to a few days in patients who have been pre-sensitized by previous exposure, and after ~ 4 – 14 days in patients who have not yet been pre-sensitized. Only in very severe delayed reactions (“Drug Reaction with Eosinophilia and Systemic Symptoms” (DRESS syndrome), Stevens-Johnson syndrome (SJS), and toxic epidermal necrolysis (TEN)) may symptoms only appear up to 4 – 8 weeks after exposure [8]. 

### 2. Clinical symptoms and correlation with chronology 

Allergies to BLAs manifest with varying degrees of severity as [10]

- IgE-mediated immediate reactions in ~ 1/3 of cases, for example with symptoms such as generalized urticaria, angioedema, shortness of breath, tachycardia, hypotension, which can be associated with itching, abdominal pain, and diarrhea. The most severe form is anaphylactic shock and even cardiopulmonary arrest [29];
- maculopapular exanthema in ~ 2/3 of cases as non-IgE-mediated T-cellular delayed reactions. Very rare severe variants are DRESS syndrome and generalized blistering skin reactions such as SJS and TEN. Caution: It is not always easy to differentiate between an immediate reaction with urticaria or angioedema and a delayed reaction with a maculopapular exanthema, which may also have an urticarial aspect in the first few days before scaling becomes visible. In case of doubt, a more dangerous drug-induced urticaria, i.e., an immediate reaction, should be assumed;
- other very rare organ reactions of different pathogenesis such as vasculitis, hemolytic anemia, other cytopenias, or acute nephritis, hepatitis (e.g., drug-induced liver injury).

### 3. Pathomechanism 

According to the Coombs and Gell classification of the pathomechanism of allergic reactions, a distinction is made between IgE-mediated immediate-type reactions (type I), the very rare cytotoxic (type II) and immune complex reactions (type III), and the frequent delayed-type allergies mediated by T-lymphocytes (type IV) [8, 11]. From the clinical symptoms and the time course (e.g., anaphylaxis 15 minutes after the first administration of a cephalosporin versus maculopapular exanthema after ~ 1 week of therapy with amoxicillin), a pathomechanism can already be assumed (IgE-mediated/type I versus T-cell-mediated/type IV). However, the underlying immune mechanism can only be confirmed by subsequent allergologic diagnostics and not by the medical history alone. 

## Differential diagnosis of BLA allergy 

Suspected BLA allergies can rarely be confirmed, especially if the reaction occurred a long time ago [5, 12]. In many cases, a suspected “penicillin allergy” in childhood is reported without more precise information being available. However, often (1) a viral exanthema is interpreted as a drug exanthema, furthermore (2) an infection-associated acute urticaria, (3) a known pharmacological side effects such as gastrointestinal symptoms due to changes in the intestinal microbiome, (4) non-specific complaints, or (5) somatoform reactions are interpreted as allergic drug reactions. Even reactions in relatives or the mere fear of an allergy (“I react to all medications”) are regarded as suspected BLA allergy [6]. 

CAUTION: Only from the clinical picture and the course of the reaction (in particular the time interval between BLA administration/intake and occurrence of the reaction) it can be estimated whether a BLA allergy is probable. This helps in deciding whether to delabel BLA, avoid BLA until further allergy diagnostics, or re-administer BLA with non-cross-reactive side chains. 

## Risk of cross-reactivity is primarily dependent on the side chain structure 

**Figure 1.**
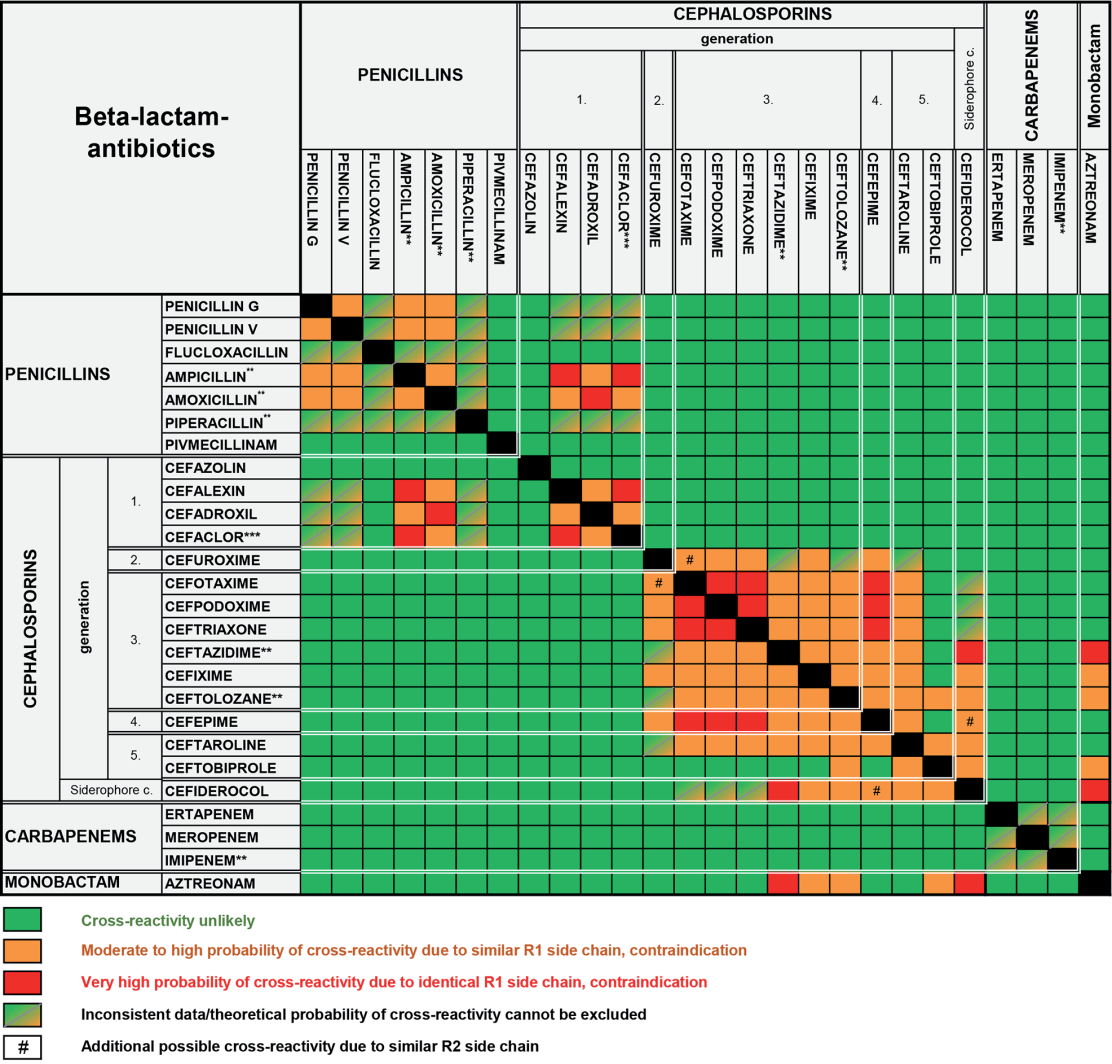
Similarity of side chain structures between β-lactam antibiotics for estimation of the most important cross-reactivities.

Knowledge of possible cross-reactivity between the BLAs is important for assessing the likelihood of a new reaction in the event of re-exposure [3, 13, 14]. This is usually based on structural similarities between the side chains (so-called side chain theory) and not, as previously assumed, on the β-lactam ring as the central basic structure of all BLAs. The probability of allergic cross-reactions between BLAs can be estimated by comparing side chain structures (Figure 1) [3, 14]. In some cases, the study data are not sufficient for a clear risk assessment. Most cross-reactions are triggered by the R_1_ side chain. The risk of an allergic cross-reaction is highest between BLAs with identical or similar R_1_ groups; substances with different side chains have a significantly lower risk. Aminopenicillins (ampicillin, amoxicillin) are considered to be almost 100% cross-reactive due to their very similar R_1_ side chain (aminobenzyl group). Some of those sensitized to aminopenicillins also show cross-reactivity with the so-called aminocephalosporins such as cefaclor, cefadroxil, and cefalexin, which can also cross-react with each other. The methoxyimino cephalosporins cefuroxime, ceftriaxone, cefotaxime, and ceftazidime also show a certain degree of cross-reactivity with each other via their side chains. However, if, for example, the suspected penicillin derivative has been reliably identified in the case of penicillin allergy, cephalosporins from the second generation onwards and cefazolin from the group of first-generation cephalosporins can be used with a very low risk of an intolerance reaction. In the case of immediate-type allergy, cross-reactivity between penicillins and cephalosporins is low at ~ 2%, between penicillins and carbapenems very rare (< 1%) and between penicillins and monobactams (aztreonam) practically unknown. Aztreonam has the same side chain as ceftazidime, but this only sometimes appears to be of clinical relevance. 

## Allergy diagnostics 

Any suspected allergic reaction in connection with a BLA should, if possible, be diagnosed during a symptom-free interval (i.e., not when the next infection occurs) with the aim to

- confirm or rule out an allergy
- identify the trigger in the event of an allergy
- detect possible cross-allergies and, if necessary, identify alternative tolerated BLAs that can be used [8].

Skin tests (skin prick, intracutaneous, and patch tests), in-vitro tests, and provocation tests should be used for allergy diagnostics, although the commercial availability of test allergens is very limited [8]. If possible, clarification should take place within the first 6 months after the onset of the drug reaction, but generally no earlier than 4 weeks after the administration of anti-allergic/immunosuppressive medication to treat the symptoms [8]. However, standard allergy diagnostics with skin testing of the person in need of treatment cannot always be carried out in a timely manner. In this respect, the possible urgent need for treatment with a BLA and the availability of an adequate alternative antibiotic for the acute infection must also be taken into account [6]. Tolerance induction is another option that can be carried out by experienced allergists, but due to the fact that BLAs with a different side chain can usually be used with minimal risk of cross-reaction, this is only very rarely necessary [15, 16]. 

## Practical procedure for suspected BLA allergy and urgent BLA therapy indication 

### General considerations 

There is an urgent need to administer a BLA if, according to the available antibiogram or due to the disease, there is a strong indication for a BLA with an expected poorer response or more side effects to therapy with another substance class. This applies in particular, if individual factors such as underlying diseases, potential side effects, or contraindications rule out the use of another substance class (e.g., cotrimoxazole for neutropenia in *Stenotrophomonas* infection). The severity of an expected reaction should also be considered. For example, after uncomplicated BLA-induced maculopapular exanthema, there is only an increased risk of recurrence of maculopapular exanthema and no increased risk of a life-threatening immediate reaction (i.e., anaphylaxis) after renewed intake. If urgently required, the recurrence of an uncomplicated exanthema can also be considered acceptable. This however is explicitly not allowed in cases where there is even a low risk of a reaction that cannot be adequately controlled with medication, such as DRESS, severe blistering skin reactions, such as SJS or TEN, and other severe organ reactions. 

**Figure 2.**
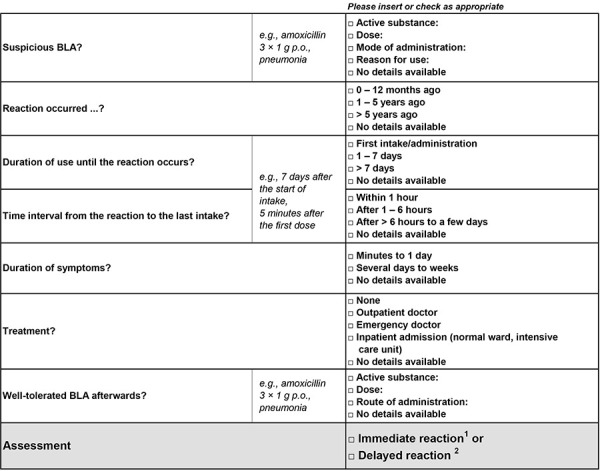
Anamnestic assessment of the reported β-lactam antibiotic allergy. ^1^Immediate reaction (type I reaction, IgE-mediated): Frequently to cephalosporins, within a few minutes to 1 hour or rarely up to 6 hours after the first administration, then lasting for several hours; ^2^Delayed reaction (type IV reaction, non-IgE-mediated): Often to aminopenicillins, after 6 hours to several days, then lasting several days to weeks. BLA = β-lactam antibiotic.

**Figure 3.**
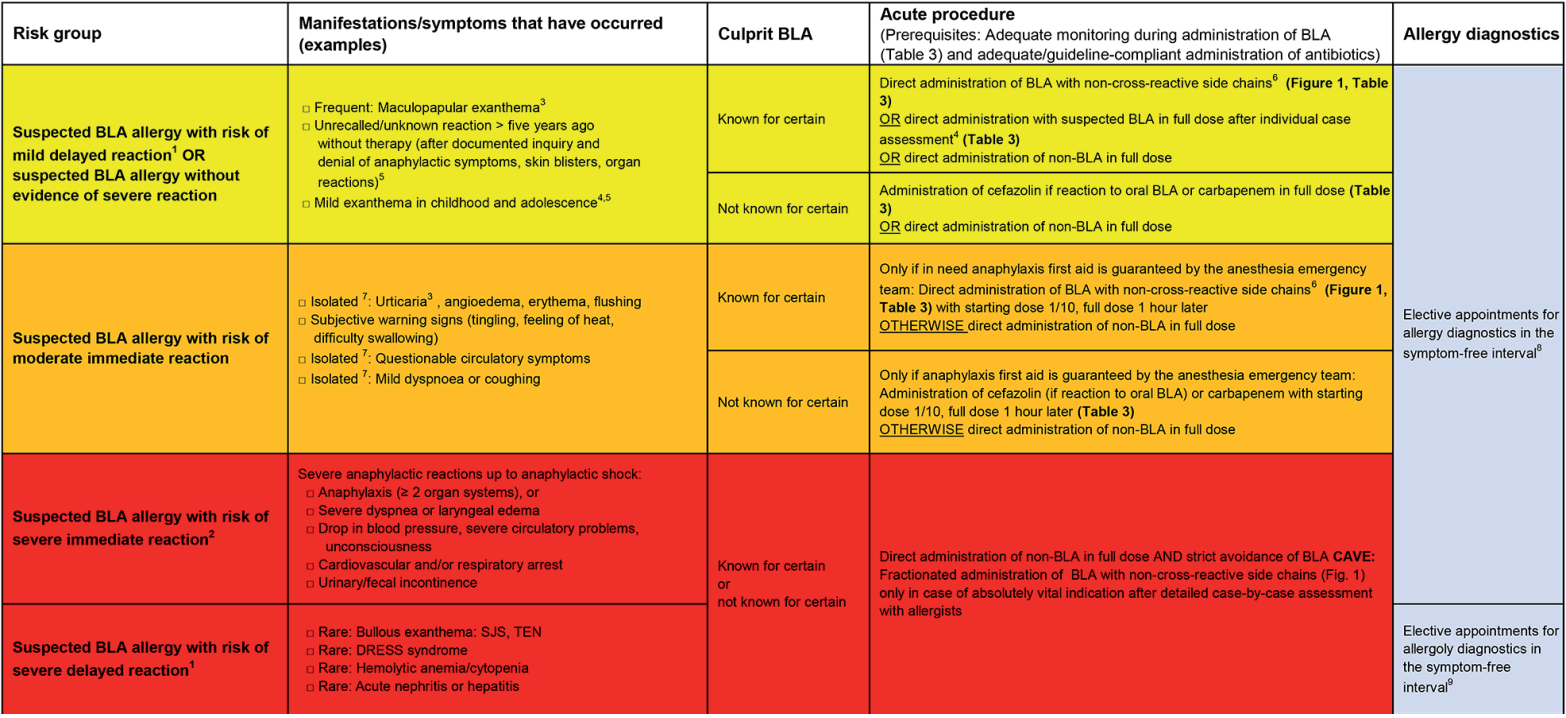
Risk stratification and practical procedure for suspected β-lactam antibiotic allergy and urgent indication of β-lactam antibiotic therapy. ^1^Delayed reaction (type IV reaction, non-IgE-mediated): Often to aminopenicillins, after 6 hours to several days, then lasting several days to weeks. ^2^Immediate reaction (type I reaction, IgE-mediated): Frequently to cephalosporins, within a few minutes to 1 hour or rarely up to 6 hours after the first administration, then lasting for several hours. ^3^“Caution: Differentiation of urticaria or angioedema versus maculopapular exanthema is not always easy, in case of doubt assume drug-induced urticaria. a) Maculopapular exanthema: Without systemic involvement/skin blisters, often measles-like appearance, itching, often extensive (trunk, arms, legs), occurrence only several hours to days after ingestion (re-exanthema within < 6 hours), the rashes remain in the same places, increase, therapy usually with topical/systemic steroids, healing with desquamation only after several days; b) Urticaria (angioedema): Red or white elevations with red surrounding area, itching, rapid onset within 1 hours after antibiotic administration, as after contact with stinging nettles, wheals heal after < 24 hours, but new wheals may appear, healing without desquamation”. ^4^Case-by-case assessment: Direct administration with the suspected BLA in full dose, e.g. children and adolescents after infection-associated exanthema, adults with low risk of mild reaction (e.g., mild exanthema in childhood or unrecalled/unknown reaction > 5 years ago without therapy after documented inquiries and denial of anaphylactic symptoms, skin blisters, organ reactions); then if tolerated: delabeling with documentation in patient file/written information for further GP care/education of patient, if necessary collection of allergy passport/card. ^5^Mild exanthema in childhood and adolescence: Without systemic involvement/skin blisters, non-urticarial, > 6 hours after ingestion, < 50% body involvement, < 7 days before healing, no hospitalization, no systemic therapy except antihistamines. ^6^If suspected penicillin/derivative is identified with certainty: Administration of BLA with non-cross-reactive side chains, e.g. cefazolin or 2nd-5th generation cephalosporins. If suspected cephalosporin is identified with certainty: Administration of BLA with non-cross-reactive side chains (Figure 1). If suspected BLA is not identified with certainty: Administration of cefazolin (if reaction to oral BLA) or carbapenems in full dose. ^7^Isolated: No other signs of anaphylaxis (e.g., only urticaria, but no other organ involvement. The occurrence of urticaria plus mild dyspnea would already be classified as anaphylaxis with risk of severe immediate reaction. ^8^“Adequate allergy treatment: Provocation testing of alternative and possibly skin test-negative suspected BLA (ideally before acute treatment indication/preoperative, always in the symptom-free interval); if provocation test is negative, delabeling with documentation in patient file/written information for further GP care/education of patient, if necessary collection of allergy passport/card”. ^9^Allergy diagnostics: Epicutaneous test possible with several suspected BLA, but very poor sensitivity. Provocation testing with the suspected trigger is not indicated, intradermal testing or provocation testing with very important drugs not suspected as triggers only after very strict indication and in individual cases. BLA = beta-lactam antibiotic; DRESS = drug reaction with eosinophilia and systemic symptoms, SJS = Stevens-Johnson syndrome; TEN = toxic epidermal necrolysis.

**Figure 4.**
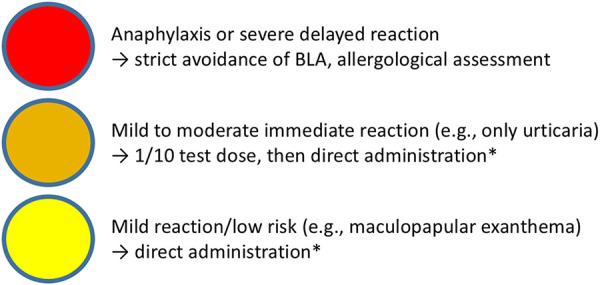
Simplified summary of the practical procedure. BLA = β-lactam antibiotic. *Administration of BLA with non-cross-reactive side chains under supervision, adequate emergency therapy guaranteed.

If a BLA allergy is suspected, a risk stratification should be carried out on the basis of the patient’s medical history (Figure 2) and any additional information (e.g., physician’s letter, allergy passport/card) (Figures 3, 4) [2, 6]. This allows to assess the probability and the expected severity for future BLA applications. If a BLA allergy can already be ruled out on the basis of this information (Table 2), the diagnosis of “penicillin allergy” can be corrected immediately (“delabeling”), even without allergological diagnostics [4]. If the presence of a BLA allergy is possible but there is no evidence of a severe reaction (e.g., uncomplicated maculopapular exanthema after BLA administration), direct administration of a full dose of BLA with side chains other than the reported trigger is possible under medical supervision if there is an urgent indication. If there is a high risk of a severe reaction to BLAs, the administration of non-BLAs to treat the acute infection is recommended. A later allergological assessment should be carried out in the symptom-free interval. The original trigger can only be delabeled if the result for the reported trigger is negative; Tolerating an alternative BLA is not sufficient. 

### Risk stratification and practical procedure 

The practical procedure depends on

- the risk for patients (Figure 3),
- whether the presumably triggering BLA is known or unknown,
- whether sufficient supervision of the treated persons can be guaranteed (Table 3). If this cannot be guaranteed, we advise against carrying out the treatment and recommend switching to an alternative non-BLA substance.

**Figure 5. Figure5:**
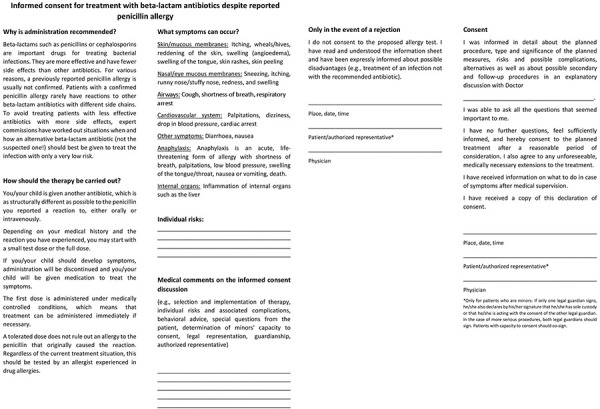
Declaration of consent for treatment with β-lactam antibiotics despite a reported penicillin allergy.

Based on the medical history (Figure 2), four groups with different risk profiles and procedures are defined (Figures 3, 4)[Fig Figure5]. A separate procedure is recommended for each of these risk profiles (prerequisites: adequate supervision when administering BLAs (Table 3) and administration of antibiotics best suited for treating the infection).

1. Suspected BLA allergy without evidence of a severe reaction OR suspected BLA allergy with risk of a mild delayed reaction (maculopapular exanthema)

**Procedure:** Direct administration of BLA with non-cross-reactive side chains (Figure 1) under adequate medical supervision (Table 3). **Caution:** In the case of reactions that cannot be classified with certainty based on the patient’s medical history, an individual assessment must be made and, in case of doubt, a higher risk must be assumed.

- Suspected BLA identified with certainty:
  - Penicillin (derivative): Administration of BLA with non-cross-reactive side chains (Figure 1) in full dose, e.g., cefazolin or 2^nd^ – 5^th^ generation cephalosporins
  - Cephalosporin: Administration of BLA with non-cross-​reactive side chains (Figure 1) in full dose
- Suspected BLA not identified with certainty:
  - Administration of cefazolin (if anamnestic reaction to oral BLA) or carbapenems in full dose

Particularly in children and adolescents after infection-associated exanthema and also in adults with only a very low risk of a mild reaction (e.g., mild exanthema in childhood/adolescence or unrecalled/unknown reaction > 5 years ago without therapy after documented inquiry and denial of anaphylactic symptoms, skin blisters, and organ reactions, the direct administration of the suspected BLA in full dose is also possible [24, 25, 27]. The first administration of a single dose should always take place under medical supervision (Table 3). In the absence of a reaction, delabeling is carried out (documentation in the patient file/written information for further GP care/education of the patient, if necessary withdrawal of allergy passport/card) if the suspected BLA was taken in full dose.

2. Suspected BLA allergy with risk of a moderate immediate reaction

**Procedure:** Direct administration of non-BLA or direct administration of BLA with a different side chain (Figure 1) under adequate medical supervision (Table 3). An emergency anesthesia team or an allergist experienced in anaphylaxis therapy must be on standby and quickly available.

- Suspected BLA identified with certainty:
  - Penicillin (derivative): Administration of BLA with non-cross-reactive side chains (Figure 1), e.g., cefazolin or 2^nd^ – 5^th^ generation cephalosporins with starting dose 1/10, then full dose 1 hour later.
  - Cephalosporin: Administration of BLA with non-cross-reactive side chains (Figure 1) with starting dose 1/10, then full dose 1 hour later.
- Suspected BLA not identified with certainty:
  - Administration of cefazolin (if anamnestic reaction to oral BLA) or carbapenems with starting dose 1/10, then full dose 1 hour later.

3. Suspected BLA allergy with risk of severe immediate reaction

**Procedure: **Direct administration of non-BLA and strict avoidance of BLAs. Even in cases of acute urgency, the administration of the suspected BLA is not normally indicated. Only if there is a vital indication and after a careful allergological examination of the individual case with a risk-benefit assessment by an experienced allergist, skin testing with the planned non-cross-reactive BLA. If the test result is negative, fractionated administration of this BLA, e.g., with carbapenems, can be considered.

4. Suspected BLA allergy with risk of severe delayed reaction

**Procedure:** Direct administration of non-BLA and strict avoidance of BLAs. In the symptom-free interval, allergological diagnostics should be carried out with an attempt to narrow down the trigger in cases where several drugs have been given, but the sensitivity of the diagnostics is often poor. Provocation tests with the suspected BLA are not indicated. Intradermal testing or provocation testing to rule out important unsuspicious drugs as the trigger should only be indicated on a case-by-case assessment after appropriate information has been provided to the patient. Even in cases of acute urgency, the administration of the suspected BLA is not normally indicated. Fractionated administration of BLA with non-cross-reactive side chains, e.g., with carbapenems, can only be considered in cases of vital indication after a detailed allergological risk-benefit assessment. 

## Conclusion 

BLAs are very important antibiotics. A high number of patients report a possible BLA allergy. This poses a risk of inadequate infection control, which can lead to increased morbidity and mortality at population level. 

When determining how to proceed, not only the risk to elicit any reaction must be taken into account, but also the presumed severity of a possible future reaction. The expected severity of the drug reaction and the controllability/effectiveness of a drug intervention are more important for risk stratification than whether there has been an immediate or delayed reaction. However, the latter has consequences for the planning of the acute procedure. If there is a risk of mild delayed reactions and an urgent indication, no reaction will be visible in the first few hours after titrated administration, so BLAs can also be given without a prior test dose. 

Most people with a suspected BLA allergy are never tested and are given an alternative antibiotic if required. However, the majority of these individuals have only a low risk of mild reactions when given alternative BLAs. To improve the situation of non-adherence to BLAs, we recommend a risk-stratified and needs-based approach with either direct delabeling, administration of BLAs with side chains of different structure under medical supervision with or without prior test dosing, or BLA avoidance. This allows most patients with a reported BLA allergy to be administered BLA substances with other side chains, if there is an urgent indication. In the DACH region (Germany, Austria, Switzerland), the sensitivity and specificity of BLA skin tests are rated as good [28]. Therefore, following acute therapy in the symptom-free interval, standard allergological diagnostics should be provided by means of targeted medical history, skin testing, laboratory diagnostics, and provocation tests with (fractionated) administration of BLAs for delabeling or identification of triggers and tolerated BLAs. 

## Authors’ contributions 

KB prepared a first draft. This was first discussed with MW, WP, BW, GW, AT, TB, BK, CQ, RT. Then the other authors contributed to the content. Finally all authors contributed in consensus finding and wrote the paper. 

## Funding 

Consensus conferences took place as webconferences. No further costs were incurred.

## Conflict of interest 

None. 

**Table 1.** Consequences of administering non-β-lactam antibiotics in the case of a reported allergy (modified from [6]).

– Delayed administration of antibiotic therapy
– Poorer efficacy and more frequent treatment failure
– Higher rate of postoperative wound infections
– Longer hospital stay and higher re-hospitalization rate
– Higher rate of transfer to an intensive care unit and higher mortality
– More frequent side effects
– Resistance due to the use of broad-spectrum and reserve antibiotics
– Higher treatment costs
– Psychological effects with lower quality of life

**Table 2.** No evidence of allergy to β-lactam antibiotics.

– Allergy documented but denied by patient
– Isolated gastrointestinal reaction: diarrhea, nausea, vomiting (to be distinguished from spontaneous occurrence in anaphylactic reactions together with other anaphylactic symptoms)
– Isolated non-specific reaction: dizziness, headache, fatigue, palpitation, rhinoconjunctivitis (often associated with fear of drug hypersensitivity)
– Delayed urticaria: occurrence > 1 day after discontinuation or persisting for days after discontinuation
– Mild exanthema: occurrence > 1 week after discontinuation
– Positive family history without personal allergy history
– Isolated generalized or localized pruritus without further skin changes and without warning signs of anaphylaxis (burning/tingling of tongue/palate, palm/sole of foot or genitals, feeling of heat, coughing, difficulty swallowing, reddening of large areas of skin)
– Anamnestic tolerance of the suspected β-lactam antibiotic administered (accidentally)
**Procedure:** Direct administration of β-lactam antibiotic without allergologic diagnostics. Direct delabeling (documentation in patient file/written information for further GP care/education of patient), collection of allergy passport/card if necessary.

**Table 3.** Institutional and personnel requirements for medically supervised exposure to β-lactam antibiotics in the case of reported β-lactam antibiotic allergy.

– Observation by healthcare professionals for at least 60 minutes after each dose increase (e.g., after administration of 10 or 100% of a single therapeutic dose), depending on the risk, under inpatient or outpatient conditions. Observation by non-medical personnel is not sufficient.
– There must be a doctor in close proximity for immediate intervention.
– In case of risk of moderate or severe immediate reaction, rapid availability of anesthesia emergency team or allergist experienced in anaphylaxis therapy
– Staff trained in dealing with anaphylactic reactions
– Availability of suitable emergency therapy equipment
– Consent of the persons to be treated and/or legal guardians (example see Figure 5)
– Informing the treated persons and/or guardians about what to do if a reaction occurs after the end of medical supervision
